# Y Chromosome Haplotypes Enlighten Origin, Influence, and Breeding History of North African Barb Horses

**DOI:** 10.3390/ani12192579

**Published:** 2022-09-27

**Authors:** Lara Radovic, Viktoria Remer, Carina Krcal, Doris Rigler, Gottfried Brem, Ahmed Rayane, Khadija Driss, Malak Benamar, Mohamed Machmoum, Mohammed Piro, Diana Krischke, Ines von Butler-Wemken, Barbara Wallner

**Affiliations:** 1 Institute of Animal Breeding and Genetics, University of Veterinary Medicine Vienna, 1210 Vienna, Austria; 2 Vienna Graduate School of Population Genetics, University of Veterinary Medicine Vienna, 1210 Vienna, Austria; 3 World Barb Horse Organization O.M.C.B., Organisation Mondiale Du Cheval Barbe, 148 Avenue de l’ALN Caroubier Hussein Dey Alger, Algiers 16000, Algeria; 4 Tunisian Horse Breeding Organization F.N.A.R.C., Foundation Nationale pour l´Amélioration de la Race Chevaline, Sidi Thabet 2020, Tunisia; 5 Royal Horse Breeding Association SOREC, Société Royale d´Encouragement Du Cheval, Haras Regional la Kasbah de Bouznika, B. P. 52, Bouznika 13100, Morocco; 6 Veterinary Genetic Laboratory, Hassan II, Agronomic and Veterinary Institute, B. P. 6202, Rabat 10101, Morocco; 7 Barb Horse Breeding Organization VFZB e. V., Verein der Freunde und Züchter des Berberpferdes e.V., Kirchgasse 11, 67718 Schmalenberg, Germany; 8 Department of Animal Breeding, University of Kassel, Nordbahnhofstr. 1a, 37213 Witzenhausen, Germany

**Keywords:** North African horse, Barb, Arab-Barb, Y chromosome, haplotype

## Abstract

**Simple Summary:**

Bred over centuries in the Maghreb region, on a corridor between the Arab and the Western world, the North African Barb horse has been touched by many influences in the course of history. The present study investigated the paternally inherited Y chromosome in today´s Barbs and Arab-Barbs collected from North Africa and Europe, with the aim to link genetic patterns and narrative history. A broad Y chromosomal spectrum was observed, as well as regional disparities among populations. Y chromosomal patterns illustrated a tight connection of Barb horses with Arabians and several other breeds, including Thoroughbreds. Besides, results depict footprints of past migrations between North Africa and the Iberian Peninsula.

**Abstract:**

In horses, demographic patterns are complex due to historical migrations and eventful breeding histories. Particularly puzzling is the ancestry of the North African horse, a founding horse breed, shaped by numerous influences throughout history. A genetic marker particularly suitable to investigate the paternal demographic history of populations is the non-recombining male-specific region of the Y chromosome (MSY). Using a recently established horse MSY haplotype (HT) topology and KASP™ genotyping, we illustrate MSY HT spectra of 119 Barb and Arab-Barb males, collected from the Maghreb region and European subpopulations. All detected HTs belonged to the Crown haplogroup, and the broad MSY spectrum reflects the wide variety of influential stallions throughout the breed’s history. Distinct HTs and regional disparities were characterized and a remarkable number of early introduced lineages were observed. The data indicate recent refinement with Thoroughbred and Arabian patrilines, while 57% of the dataset supports historical migrations between North Africa and the Iberian Peninsula. In the Barb horse, we detected the HT linked to Godolphin Arabian, one of the Thoroughbred founders. Hence, we shed new light on the question of the ancestry of one Thoroughbred patriline. We show the strength of the horse Y chromosome as a genealogical tool, enlighten recent paternal history of North African horses, and set the foundation for future studies on the breed and the formation of conservation breeding programs.

## 1. Introduction

The history and origin of the North African horse have been long debated [1]. Still, there is no confirmation of horses inhabiting Africa, or evidence of domesticated horses roaming around the continent in early prehistoric time, but discussions about an “Equus Algericus” found near Tiaret (Algeria) still remain [1,2]. However, historical and archeological findings indicate that the introduction of the domesticated horse to North Africa was likely in the late second millennium BCE, via several routes following human migrations and conquests (e.g., through Strait of Gibraltar or Egypt) [3,4,5]. 

The origin stories of the North African Barb horse lead off the Barbary coast in the Maghreb region (today’s Algeria, Tunisia, Morocco), hence the name “Barb”. Foremost, Numidian horses and their crosses are especially discussed as founders of the breed [6,7]. Complex patterns of human and horse migrations in the North African region peaked around the 7th century, concurrent with the Muslim conquests [8,9]. Later, during the occupation of the Iberian Peninsula by the Moors, from the early 8th to the late 15th century, migrations between North Africa and Iberian Peninsula were frequently ongoing [3,8,10,11] and the influence of the North African horses onto Iberian stocks was substantial [12,13].

Numerous myths exist on the multilayer history of the Barb horse, for example, phenotypic traits relate the discussion about the progenitors to Mongolian horses, as well as the rare light-colored (cream-gene) and piebald (sabino) horses, corresponding to the Turkoman and the Akhal Teke breed [1,14]. Barbs had a prominent role as war horses and for breeding in Europe [1,12]. Notably, Barb horses were used in the Punic wars (264–146 BCE) that were fought between Romans and Carthage, and later exported to Europe by Carthaginian conquests [14]. Likewise, more heavy horses were introduced to the Maghreb region first by Romans (from 146 BCE) and later in the 17th century by Louis XIV [7]. However, after the 18th century, breeding declined dramatically because Barb horses were no longer used for the military cavalry, due to the shift of military tactics that began in the 19th century [1,15]. More recently, from the end of the 19th century onwards, cross-breeding of North African and coldblooded horses from France resulted in the “Breton-Barb”. In addition, crosses of the Barb horse with Thoroughbreds, Anglo-Arabs, and French Trotters in North Africa were reported [1,12,15]. Above all, systematic cross-breeding with Arabian horses founded the “Arab-Barb” breed in the Maghreb region. In the 20th century during both world wars, French colonial cavalry and later also under Rommel´s regime, captured Barb horses and this contributed to their diffusion throughout Europe [16]. Moreover, from 1965 onwards, the African horse sickness significantly reduced North African Barb horse populations and prevented horse export to Europe for over ten years from Algeria [17], and from Morocco during 1987–1991 [18].

In 1987, the “Organisation Mondiale du Cheval Barbe (OMCB)” was founded to preserve the purebred Barb horse and its cross populations (“derivates”), especially the Arab-Barb horse [19]. The OMCB is nowadays recognized as a competent authority for setting up the breeding programs. Breed registries were only recently established for Barbs and Arab-Barbs in the Maghreb region (1886 in Algeria, 1896 Tunisia, and 1914 Morocco) [1,14]. Since then, the studbooks remained open so that phenotypically classified horses can be entered retrospectively, even if no known ancestry can be proven (defined as “*Inscription à Titre Initial*”, “ITI”) [20]. Additionally, European registries are established (in France in 1989, Germany 1992, Switzerland 1993, and in Belgium from 1992–2017) and their studbooks are closed. Barb horses and the Arab-Barb horses are separated in different studbooks or studbook sections according to the OMCB stud-book regulations. The stud-book section for Arab-Barbs is still open for Arab/Barb crosses as well as crosses of Arab-Barbs with either Barbs or Arabs. All over, studying ancestry and breeding histories in North African horses via pedigree documentation is limited. 

The census population size in the Maghreb countries is about 5500 for Barbs and 180,000 for Arab-Barbs [21,22]. Out of those, 1800 Barbs and 26,000 Arab-Barbs are registered in studbooks. In contrast, the European subpopulation constitutes about 2800 Barbs and 4000 Arab-Barbs, out of which 520 and 440 horses (Barbs and Arab-Barbs, respectively) are registered for breeding in the OMCB recognized studbooks. They produce about 160 foals per year [21,22]. The breeding programs for Barbs and Arab-Barbs are mainly based on characteristic phenotypic traits, robustness, and behavior rather than uniform breeding goals. Today, these horses are used for “Fantasia” (also known as “*Tbourida*” in Morocco and “*Mchef*” in Tunisia) a traditional equestrian war game dating back to the 16th century, as well as for agricultural work, carriage, riding, dressage, and equestrian art, as well as racing (only Arab-Barbs) in North Africa [1,12,19]. In Europe, they are used as leisure horses, for endurance-riding, historical dressage, jumping, and working equitation [1,16].

According to the diverse use and breeding areas, the North African Barb and Arab-Barb horse populations are characterized by broad phenotypic variation [1,22,23]. Within the Arab-Barbs, this strongly depends on the percentage of Arabian ancestry [24,25]. Investigation of blood group markers, protein, and DNA polymorphisms in North African subpopulations showed a pronounced genetic variation within the Barbs and the Arab-Barbs. Private alleles and high levels of heterozygosity were noted, however, no significant genetic differentiation was observed between Barb and Arab-Barb populations [26,27,28,29]. Likewise, apparent phenotypic differences distinguish the purebred Barb horse from the Arabian horse [1,23,25,30,31]. Microsatellite analysis showed similarities between the Arab-Barb and Arabian horses and a clear genetic separation of both breeds from Thoroughbreds [27,28,29]. The maternally inherited mitochondrial DNA showed close genetic relationships between Iberian breeds and Barb horses [11,32]. Nevertheless, the relationship between the North African Barb and the Arab horse has been continuously debated, till today [33]. 

A prominent genetic marker for inferring the ancestry of populations is the non-recombining, male-specific region of the Y chromosome (MSY). The MSY is inherited exclusively from the father to his sons and thus MSY haplotypes (HTs) mirror the paternal lineages in a population. MSY analysis is best established in humans where it is widely used in population genetics, genealogical research, and forensics [34,35,36]. In domestic horses, the MSY was long excluded from population genetic studies due to the lack of informative sequence polymorphism (reviewed in [37]). Nevertheless, a stable MSY HTs topology based on slowly evolving biallelic markers was constructed by mapping next generation sequencing (NGS) data to a 6.5 Mb horse MSY draft reference [38]. The MSY HTs of domestic horses are clearly distinct from those in the extant Przewalski’s horses. The most pronounced MSY signature among domestic horses is the ~2000-year-old “Crown” haplogroup (HG), recounting various breeds from Central and South Europe, East Asia, North and South America [38,39]. It was proposed that the dominance of the Crown HG is a hallmark of the recent breeding influence of stallions of Oriental origin [38,40]. The crown topology supports the hypothesis [41,42] that only a limited number of stallions contribute to today´s horse population. Only some Asian horses [43,44] and Northern European breeds (e.g., [45]) seemed to be unaffected by the recent Oriental introgression, and thus kept their autochthonous HTs outside the Crown (“Non Crown”). Within the Crown, three HGs were defined (H, A, and T) and the HT signatures of three English Thoroughbred founders [38], as well as Arabian patrilines [39] were recently successfully delineated. 

In horses, MSY analysis can unmask patrilines that contributed to a breed; thus, impart motifs of their male demography, and shed light on complex breeding histories. In this study, we investigated MSY HTs in North African Barb horses with the aim to link Y-chromosomal patterns to narratively known historical events. We hypothesize that the long-lasting input of foreign blood and complex migrations in the Maghreb region will be mirrored in their MSY HT spectrum. In addition, due to indigenous origin, regional and less intensive selection strategies [1], we might detect the preservation of autochthonous HTs in some North African horses’ patrilines.

## 2. Materials and Methods

### 2.1. Sample Set

Biological samples were collected from 119 males, of Barbs (n = 84) and Arab-Barbs (n = 35) in Morocco, Algeria, Tunisia, and the European subpopulations. To ensure that many patrilines were represented in the dataset, pedigree information (available for 86 horses), provided by breeding authorities and associations, was considered in the sampling strategy as previously described [39]. Hence, oversampling of relatives was averted from the dataset by keeping six males per foundation sire at maximum. Additionally, we included 33 randomly sampled horses without pedigree information (10 European and 23 North African samples) to complement and capture population variation beyond documented patrilines. The dataset including individual male tail line information for ancestors born prior to 1990 is given in a string format in Table S1.

### 2.2. MSY Genotyping

We inferred MSY haplotype spectrum of 119 samples according to the previously reported horse Y phylogeny [38,39]. For genotyping, we created a downscaled HT structure based on 65 selected HT-determining variants as markers (61 SNVs, 3 short Indels, and 1 microsatellite, see Supplementary Table S2). The resulting tree served as the backbone and samples were placed onto branches of the tree via MSY marker screening. 

For variant screening, genomic DNA was isolated from hair roots or blood with the nexttec^®^ DNA Isolation Kit. The DNA was then diluted with TE buffer to the uniform concentration of 5 ng/μL. Genotyping of variants was performed using competitive allele-specific PCR SNV genotyping assays (KASP™, lgcgroup.com (accessed on 2 July 2021)), following the standard protocol on a CFX96 Touch™ Real-Time PCR Detection System. Samples with known allelic state were included as positive controls, while DNA from females and non-template controls were used as negative controls. Information on variants (coordinates on LipY764, alleles, and flanking regions) are published in [38,39].

Genotyping of the amplicon length of the tetranucleotide microsatellite fBVB (GATA14/GATA15) was performed on an ABI 3130xl Genetic Analyzer, as previously described [38]. In synopsis, for the fragment analysis, one PCR primer was tagged with FAM fluorescent dye (fwd_FAM: ACAACCTAAGTGTCTGTGAATGA; rev: CCCAATAATATTCCACTGCGTGT, expected amplicon length 204 bp). PCR was carried out in a 20 µL reaction volume containing 0.4 µM of each primer. The reaction temperature was increased to 95 °C for 5 min for initial DNA denaturation, followed by 35 cycles of 30 s at 95 °C, 40 s at 58 °C annealing temperature and 40 s at 72 °C, and a final extension step of 30 min at 72 °C. Finally, GeneMarker^®^ was used to size the alleles relative to the internal size standard. 

**Figure 1:**
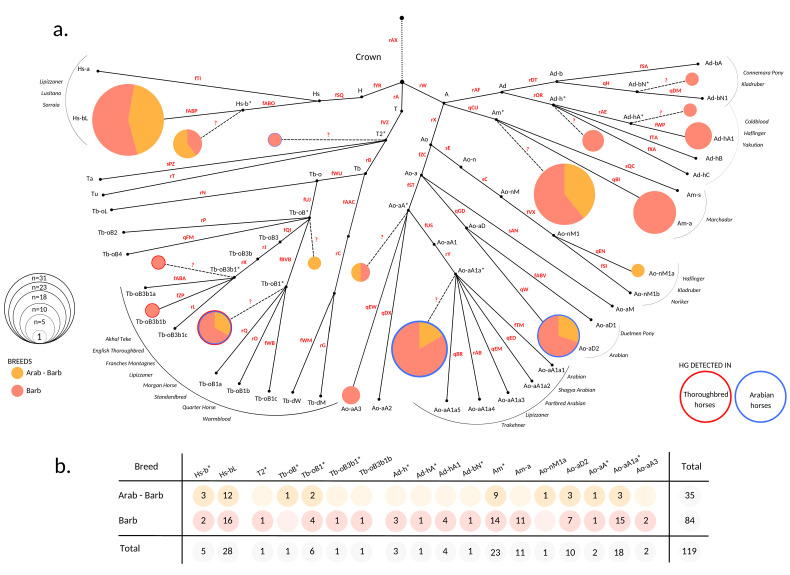
MSY haplotype spectra of North African horses. (**a**) HT frequency plot based on the MSY tree after [38,39]. HT determining variants used to construct the downscaled tree for genotyping are denoted on branches in red. Additional information is given in Supplement, Table S2. Clustering of 119 North African horses based on genotyping result is illustrated as pies. Pie radiuses are scaled to the number of allocated individuals and colors of the portions correspond to different breeds. HG names are labeled accordingly. HTs located on internal nodes are denoted with an asterisk (*) and trailed with dashed lines that originate from corresponding internal nodes. Unascertained variants that would determine * HTs are denoted with question marks (?). HTs framed with blue and/or red borders denote that they were detected previously in Arabian (blue border) and Thoroughbred (red border) horses [38,39]. Non-colored points express HTs that were not detected in the North African sample set. Gray list on the sides of the network indicates the breeds the HTs were previously reported [38,39,40]; (**b**) Number of individuals that allocate within detected HTs. Sample information details are given in Supplement Table S1.

Genotyping was conducted in a consecutive manner by first testing the Crown determining variant rAX. If a sample carried the derived C-allele for this variant, allocation of the sample into main Crown HGs H, A, or T was conducted by testing markers fYR, rW, and rA. Each sample was then typed for the markers determining the substructure of the HG it clusters into. We then merged the genotyping information of all tested variants and imputed the allelic state of markers that were not tested or detected in the sample set according to the previously published HT structure [38,39] (see Figure 1 and Supplementary Table S2). We generated a median-joining HT network with program Network 10.2 [46] and redrew it as a HT frequency plot (Figure 1) in Canva Pro (https://www.canva.com (accessed on 29 June 2022)). Pie charts were drawn and scaled to the respective number of samples with RStudio version 4.0.3. [47].

## 3. Results

To investigate the MSY HT spectra of North African horses, 119 males representing 84 Barbs and 35 Arab-Barbs were genotyped. The results showed that all samples allocated into the Crown HG. In total, we distinguished 18 HTs and all three previously defined Crown HGs (A, H, and T) were represented in our sample set. The broad Crown MSY HT spectra was comparable in Barbs and Arab-Barbs (Figure 1). This is in contrast to patterns in other today`s breeds [38,39] that showed distinct clustering on the tree. Remarkably, only half of the males analyzed carried defined HTs, whereas 61 males got placed at internal nodes of the backbone topology (See Figure 1 and Table S2). The samples allocated at inner nodes are marked with an asterisk (*) in their HT identifier and distinguished with dashed lines in Figure 1. For instance, the sample that allocates into Tb-oB* HT carried the derived allele for the fUJ marker and was placed onto the branch Tb-oB, but it carried the ancestral allele at the markers determining subsequent HTs in our backbone tree (rP, qFM, fQI, and fBVB). The inner node clustering of samples occurs when the HT of the horse is not represented by the tree due to ascertainment bias, and only the HG and the branching point could be determined.

More than half (56%, n = 67) of the analyzed individuals are distributed across two HGs, Am (n = 34) and Hs-b (n = 33), respectively (see Figure 1). Other than North African horse, these HGs were so far only detected in some South American and Iberian breeds [35,36]. Besides, we observed grouping of 28 (24%) males into Ao-aA1a* and Ao-aD2 HTs. Those HTs were designated recently as signatures for Arabian horses [39]. The arrangement of the internal branching points in the strictly hierarchical MSY HT tree topology reflects the emergence of the mutations over time. Hence, the HTs Ao-aA* (n = 2) and Ao-aA3 (n = 2) can be interpreted as hints to earlier introduced lines of presumably Arabian origin, that evolved and are still preserved in the North African Barb horse. We further aggregated ten males in the Tb HG. Among those, two males clustered onto early branching points (T2* and Tb-oB*) and six were allocated in the HT Tb-oB1*. This HT was previously reported in Akhal Teke, Turkoman, Thoroughbreds, as well as Arabian horses [38,39,40]. The Tb-oB1* in North African horses can be explained as the recent influence of stallions from that region. Noteworthy, we detected Tb-oB3b1*, the HT basal to the HTs detected in the progeny of the Thoroughbred´s founder sire ‘Godolphin Arabian’, which are (Tb-oB3b1a/b/c) [38], in a Barb breeding stallion from Morocco. We found the signature of recent influence of Warmblood or Thoroughbred in a single horse from France carrying Tb-oB3b1b, but did not observe the typical Thoroughbred and Trotter HGs Tb-dW and Tb-dM [38,40]. Moreover, ten males carried HGs, which are today mainly found in Coldbloods and European Ponies [39,40], namely Ad-h (8), Ad-b (1), and Ao-n (1). Here, we again observed well resolved HTs (for example Ad-hA1), as well as earlier branching off HTs (Ad-bN*, Ad-h*). 

**Figure 2:**
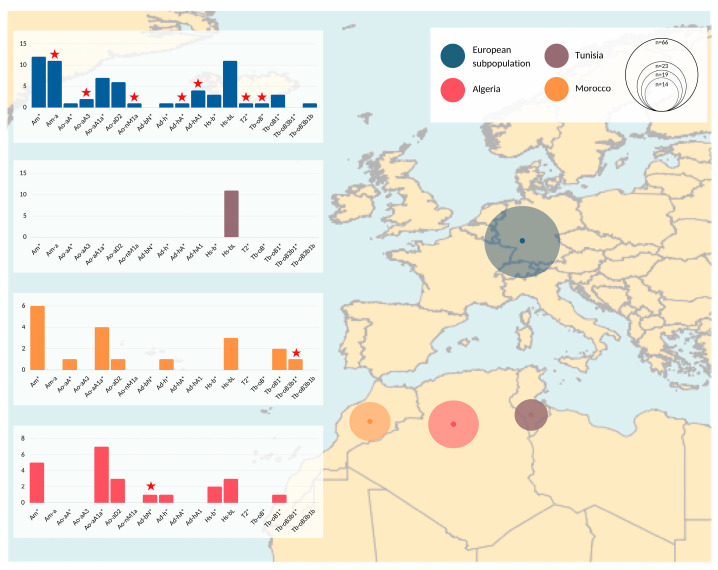
Geographical representation of MSY haplotypes. Populations analyzed are denoted with different colors and circles on the map correspond to the sample size. Details are given in Supplement, Table S1. Summary information of genotyping results and regional differences are visualized with bar plots. The x axis on the bar plots corresponds to detected HTs, while the y axis indicates number of samples that correspond to each of the bars (HTs). The samples assigned to inner nodes are marked with an asterisk (*) in their HT identifier. Red stars indicate HGs that were found exclusively in the corresponding subpopulation (e.g., seven HGs denoted with red stars in the European subpopulation are found only among samples collected in European countries, and were not observed in samples from Maghreb countries).

Roughly half of our sample set was collected in Europe and the other half in Algeria, Morocco, and Tunisia (see Figure 2 and Table S1). The samples from Algeria and Morocco clustered in 8 HTs each. The European samples clustered into 16 HTs. Seven HTs were represented only within this population group in our sample set, noting that two HTs, Ad-hA* and Tb-oB*, were detected in ITI horses directly imported from, respectively, Algeria and Morocco. The broad HT spectrum detected in samples collected in Algeria, Morocco, and Europe was not corroborated by Tunisian data. All collected Barbs (n = 9) and Arab-Barbs (n = 2) from Tunisia and all males exported from Tunisia to Europe (see below) allocated into HT Hs-bL.

**Figure 3:**
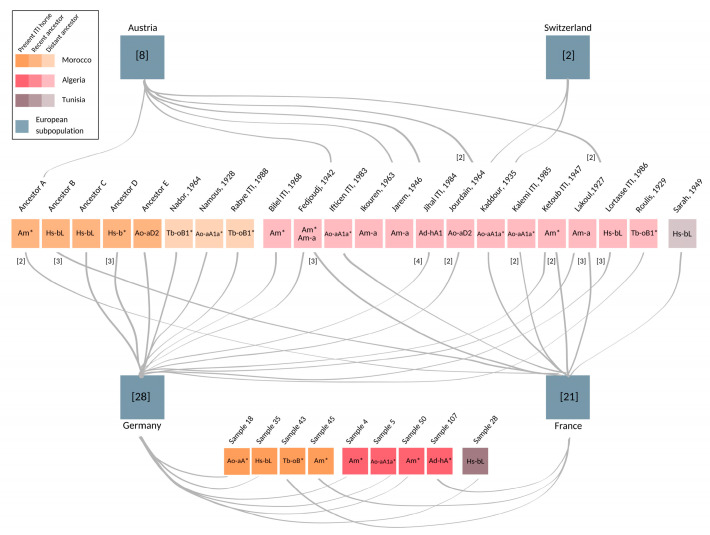
Maghrebian roots of European stallions. Fifty-nine European individuals, who were imported or their patrilines trace back to North Africa, are grouped based on their current registry (blue boxes). Number of horses included from Austria (n = 8), Switzerland (n = 2), Germany (n = 28), and France (n = 21) are denoted in square brackets. The paternal ancestors of the sampled individuals several generations back in time, as well as present individuals imported to France and Germany, are shown as colored boxes. The opacity of boxes indicates temporal layers whereas the brightest boxes on the bottom are present ITI horses, followed by recent ancestors born after 1990 (Ancestor A, B, C, D, and E), and lightest colored distant ancestors, in the middle. Name and year of the birth of ancestors is given for distant ancestors. MSY HTs, revealed from the European progeny, are shown within each stallion’s box. HT identifiers attributed with asterisk (*) denote inner node clustering. The grey lines connect the stallions with their descendants sampled in the respective European countries. Numbers in the brackets and adjacent to the left side of connection lines represent the number of descendants from each stallion found in European samples, if different from one. Pedigree details and full list of samples are given in Supplementary Table S1.

Among the 66 European samples, nine were collected from horses imported from Algeria (4), Morocco (4), or Tunisia (1). Complementing pedigree information was available for another 56 European samples (see Supplementary Table S1). This documentation reveals that the majority, namely 50, of the European males also directly trace back paternally to Maghrebian stallions exported from Algeria, Morocco, and Tunisia to Europe during the last 35 years (see Figure 3 and Supplementary Table S1). Hence, only seven out of the 66 males in the European dataset could not be linked explicitly to a hitherto known Maghrebian line from documented records. Among those, five individuals descend from four stallions, who were inscripted as ITI in the course of the foundation of the French studbook in 1989. For one sample, we had no pedigree information, and for one founder, the country of origin was unknown (see Supplementary Table S1).

Overall, the full dataset (n = 119) included 33 individuals without pedigree information (10 European and 23 horses from Maghreb) and the HT pattern in horses with and without pedigree were comparable (Supplementary Table S1).

## 4. Discussion

The significant role of North Africa, as a transit route, during the Islamic conquest and migratory movements between countries of the region [3,14], raised our interest on the Y chromosomal signature of North African Barb horses. While the MSY HT signatures of the Arabian and the Thoroughbred and their recent breeding influences are well described [38,39], the historically impactful North African horse remains enigmatic. We applied MSY haplotyping in a total of 84 Barbs and 35 Arab-Barbs, whereas half of our samples were collected in Europe and the other half in Algeria, Tunisia, and Morocco (see Figure 2 and Supplementary Table S1) and hypothesized that the MSY signature will mirror the variety of encountered influences. On the other hand, due to the documented indigenous origin and regional subgroups in North Africa, we expected partial representation of autochthonous patrilines.

The results of haplotyping indicate that no distantly related lineages were retained in the collected sample set since all horses clustered within the Crown HG. In line with previously determined time to the most recent common ancestor [38], we can state that the MSY of North African horses only reflects the last 1500 years of population history. The sole detection of the Crown mirrors influences of Oriental stallions [40]. Interestingly, we report a broad HT spectrum of North African horses across the Crown HGs (18 HTs). However, unlike other breeds (like Arabians and Thoroughbreds), for which it was possible to pin-point characteristic HGs and even discriminate discrete sublines with the use of pedigrees [38,39], the diffused HT distribution result in a tangled MSY footprint of North African horses. The observed preservation of a variety of HTs may be the consequence of less intensive selection on males and different breeding goals in North African regions. Interestingly, MSY results were comparable in Barbs and Arab-Barbs. This verifies the inter-crossing and gene flow till today between the North African horse populations, as already depicted with autosomal genetic markers [27,28,48].

However, the broad HT spectrum was not supported from Tunisian samples (n = 11), where all nine Barbs and two Arab-Barbs were monomorphic, carrying a single HT (Hs-bL) (Figure 2). This may demonstrate geographical disparities in breeding goals, supported by regional differences reported in the phenotype [1,23,30], as well as genetic spatial interpolation (e.g., [27]). In contrast, the analysis of microsatellites resulted in similarity of Moroccan and Tunisian Barb horse populations [29]. Regional differences are highlighted when we compare the HTs represented in Europe to the Maghreb region. Samples from European countries harbored seven HTs that were not represented in the samples collected in North Africa. Three of those patrilines were imports from North Africa after 2001 and four HTs trace back to the French ITI-inscriptions in 1989. Their private HTs may be explained with geographical separation of former exports to France. Additionally, we found two HTs each private for Moroccan and Algerian Barbs (Tb-oB3b1* and Ad-bN*, respectively). Compared to Tunisia, we observe similar MSY patterns in Europe, Morocco, and Algeria. One explanation for greater similarity of HTs among the latter three could be the tighter historical connection between those regions (export especially of ITI horses from Morocco and Algeria to Europe as seen in Figure 3). Nevertheless, we should interpret these findings with caution since it is possible that despite our efforts to collect a representative sample set from the Maghreb, the numbers of horses available from Tunisia was lower (n = 11). Hence, we could have underestimated HT diversity in that region.

All we see today is what is left throughout the time, and the MSY is a perfect tool to trace patrilines that shaped present populations. The relationship between the North African Barb and the Arabian horse has been continuously debated [33]. We noted a prominent clustering to Ao-aA1*, a HG previously detected in Arabian lines [39]. The detection of numerous Arabian HTs demonstrates the significant influence of Arabian stallion lines in Barbs and Arab-Barbs. A clear Arabian signature was visible in about a third of the analyzed samples. For the Arab-Barbs, the results are not surprising since the breed is based on Barbs refined with Arabians [49]. On the other hand, assignment of “purebred Barbs” to Arabian HGs may reflect, as hypothesized, recent historical migratory movements resulting in admixture, because the studbooks for the “purebred Barbs” are still open in North Africa and stallions without pedigrees are used for breeding. 

Two third of the analyzed samples (85 North African horses) did not carry the Arabian signature HTs. Particularly interesting is that among those were 27 Arab-Barbs. In addition, we detected indications of recent upgrading with European Coldbloods in four males (Ad-hA1), which could be explained with the discussed influence of Coldblood stallions imported to North Africa [12]. Moreover, only a single individual carried an unambiguous sign of Warmblood or Thoroughbred male ancestry (Tb-oB3b1b) [38].

Barbs were used for upgrading and formation of many modern breeds [12,50]. There have also been reports on their contribution to Thoroughbreds, Anglo-Arabs, and French Trotters. Interestingly, North African horses´ HTs share branching points basal to the HTs observed in many todays Coldblood, British and European Ponies (Figure 2; detected in Ad-h, Ad-b, and Ao-n HTs) [39,40], which can be interpreted as the influence of the North African horses had on those breeds further back in time. Deeper investigation is needed to validate the proposed correlation.

A particularly remarkable finding was the observation of the HT basal to the HTs spread through the Godolphin Arabian sire line (Tb-oB3b1*) [38] in a Barb horse. There is still controversy about the ancestry of Godolphin Arabian, one of the foundation sires of the English Thoroughbred (exported from Tunisia to France in 1731). He is often referred to as Godolphin Barb due to his North African origin [51] and phenotypic marks different from the Arabian horse [1,12,13]. The MSY finding, namely detection of the basal Godolphin Barb HT in a Barb horse, again fuels the discussion on the origin of Godolphin Arabian, whether he was a Turkoman stallion with partial Arabian blood [52] or corroborates the hypothesis that the Tb-oB3b1 HG made its way into the Thoroughbred via the Barb horse [1,49]. 

When we look further back in time, from the Carthaginian civilization in the 1st century and Muslimic conquests in the 7th century to recurrent migrations with Iberian Peninsula (8th to 15th century), North Africa served as a main migratory route for many cultures [3,14]. Every culture that was present in the region could have left footprints in the horses’ genomes, and this was depicted on the MSY. Notably, influence from the Middle East could be attributed to inner clustering of individuals to Ao-aA* and Tb-oB*, as well as allocation to Ao-aA3, Ao-aD2, Tb-oB1*, and T2* HGs. This grouping may indicate previously discussed influence of the ancestors of Arabian and Turkoman lineages on North African horses. 

From the viewpoint of interactions between the North African regions and the Iberian Peninsula, previous research delineated homogenous mtDNA patterns within ancient [53] and modern [11,32] horse populations in Iberia and North Africa. Particularly, it is speculated that Barb and Iberian horses have a common origin [54]. A great number of North African horses that were analyzed [32] shared mtDNA HTs reported in South American and Iberian breeds. Accordingly, we note that two highly frequent HGs (Am and Hs-b) represented in our dataset also allocate Iberian and New World horse breeds, like Marchador (Am), Lusitano, and Sorraia (Hs-b) [39]. Iberian and New World breeds are not yet comprehensively studied for their MSY HTs, but the preliminary joint clustering could reflect the gene flow and recent shared ancestry of North African Barb and Iberian horses. However, to fully explain the assumed shared ancestry further back in time, as well as the magnitude of gene flow, and indices on New World horses´ ancestry, we should complement the dataset with additional Iberian and New World horse breeds in the future. Early separated populations, like the West African Barb, the Spanish Barb (USA), and South American breeds, as well as ancient DNA samples from the Maghreb, should enlighten another chapter in horse history. Additionally, basal allocation of samples in the tree topology and underrepresentation of private HTs (Figure 1) raises a discussion on technical limitation of our analysis. The MSY backbone topology was constructed based on the ascertainment panel from [39], where five Barbs and one Arab-Barb were sequenced. However, it seems this is still insufficient, and more individuals need to be sequenced in order to clarify MSY signatures private for North African horses, in particular in HGs Hs-b, and Am. 

Overall, North African horses retained the print of the “early Oriental influence” starting with the Muslim conquests. With the observed broad HT spectrum, these horses could be a reservoir of genetic diversity—although their population is small. Further investigation of additional males, especially from the Maghreb regions, is needed to precise influential patrilines, as this is of particular practical interest for breeding. The MSY patterns should be considered together with autosomal markers, as well as mitochondrial DNA, while constructing necessary conservation breeding programs, to preserve the North African Barb horse.

## 5. Conclusions

Our study highlights the value of the Y chromosome analysis for horse population genetics and for the first time, enlightens recent paternal population history of the North African Barb horses. Obtained MSY HT spectra point to, on the one hand, that stallions were probably wide-spread hundreds of years preceding the formation of modern horse breeds, and on the other hand, indicate the impact on historical migrations and recent upgrading. However, with our approach, it is at the moment not possible to pin-point where and when the ancestors of North African Barbs came from, as well as the direction of gene flow. Future analysis on ancient DNA, as well as inclusion of more diverse Barb populations, are essential for dating of the origin of HGs, and exact inference of genetic influences. In addition, the ascertainment bias represented with HTs that are not fully resolved indicates that, even though the Crown is well described, there is still a lot left to explore in future research. Finally, our findings enhanced our knowledge of paternal ancestry of the breed and provided basis for future work and establishment of conservation breeding programs.

## Data Availability

Not applicable.

## Supplementary Materials

The following supporting information can be downloaded at: https://www.mdpi.com/article/10.3390/ani12192579/s1, Table S1: Sample set information; Table S2: Genotyping results; Table S3: Variant information.
